# TECHNIQUES AND MATERIALS TO TREAT SHOULDER PATHOLOGIES BY ARTHROSCOPY: A SURVEY IN MEMBERS OF THE BRAZILIAN SOCIETY OF SHOULDER AND ELBOW SURGERY

**DOI:** 10.1590/1413-785220243201e283711

**Published:** 2025-04-07

**Authors:** GUILHERME MACILLO CORREIA, GUSTAVO DE MELLO RIBEIRO PINTO, RODRIGO CHAUKE REZENDE, CRISTIANO NABUCO DANTAS, MARCELO COSTA DE OLIVEIRA CAMPOS, GILBERTO ZINN SCHÜTZ

**Affiliations:** 1 Universidade do Estado do Rio de Janeiro. Hospital Universitário Pedro Ernesto, Rio de Janeiro, RJ, Brazil.

**Keywords:** Rotator Cuff Injuries, Shoulder Dislocation, Acromioclavicular Joint, Arthroscopy, Bankart Lesions, Lesões do Manguito Rotador, Luxação Glenoumeral, Articulação Acromioclavicular, Artroscopia, Rupturas de Bankart

## Abstract

**Objective:**

To evaluate the preferences of shoulder and elbow surgeons from the Brazilian Society of Shoulder and Elbow Surgery to treat rotator cuff injuries, glenohumeral instability, and acromioclavicular dislocations considering a current and an ideal scenarios.

**Methods:**

A nationwide survey included 314 specialists who answered a 20-question questionnaire on treating shoulder pathologies.

**Results:**

This study included 314 specialists. Most (96%) perform rotator cuff repair arthroscopically and 74% use metallic anchors as a fixation method. In open surgery, most specialists reported using anchors (75%) instead of transosseous sutures. In treating glenohumeral instability via arthroscopic Bankart repair, 86% used three or more fixation anchors and 87%, bioabsorbable anchors. In Latarjet cases, 57% used cannulated screws. In treating acute acromioclavicular dislocations, 88% used the open route. Regarding fixation, 70% chose high-resistance wires; 65%, anchors; and 61%, Kirschner wires.

**Conclusion:**

The current Brazilian scenario has limited availability of ideal instruments and materials so specialists can treat shoulder pathologies. However, specialists’ preferences agree with the latest medical literature. ***Level of evidence V, Expert opinion.***

## RESUMO

**Objetivo:**

Avaliar as preferências dos cirurgiões de ombro e cotovelo da Sociedade Brasileira de Cirurgia do Ombro e Cotovelo em relação ao tratamento das lesões do manguito rotador, instabilidade glenoumeral e luxação acromioclavicular, considerando os cenários atual e ideal.

**Métodos:**

Uma pesquisa nacional envolveu 314 especialistas que responderam a um questionário com 20 perguntas sobre o tratamento de patologias do ombro.

**Resultados:**

Este estudo incluiu 314 especialistas. A maioria dos especialistas (96%) realizam o reparo do manguito rotador pela via artroscópica e 74% utilizam âncoras metálicas como método de fixação. Na cirurgia aberta, a maioria relatou utilização das âncoras (75%) ao invés de suturas transósseas. No tratamento da instabilidade glenoumeral através do reparo artroscópico de Bankart, 86% utilizaram 3 ou mais âncoras de fixação, com 87% utilizando âncoras bioabsorvíveis. Nos casos de Latarjet, 57% utilizaram parafusos canulados. No tratamento da luxação acromioclavicular aguda, 88% realizaram o procedimento pela via aberta. Para a fixação 70% escolheram os fios de alta resistência, 65% as âncoras e 61% os fios de Kirschne.

**Conclusão:**

No cenário brasileiro atual, há limitações na disponibilidade de instrumentais e materiais ideais para o tratamento de patologias do ombro pelos especialistas. No entanto, as preferências dos especialistas estão alinhadas com a literatura médica mais recente. **Nível de evidência V, Opinião do especialista.**

## INTRODUCTION

The Brazilian Society of Shoulder and Elbow Surgery (SBCOC) currently has about 1200 members throughout Brazil. The country, with continental dimensions and very different socioeconomic realities, offers surgeons many choices regarding the technique and materials to surgically treat the main shoulder pathologies.

Rotator cuff injuries feature among the most common upper-limb orthopedic conditions, showing a prevalence of 10% in older adults aged over 60 years.^1^

A study carried out in the United States showed the growth in the number of rotator cuff repairs from 2007 to 2016 in the age group ranging between 50 and 64 years. ^(^ ^2^

The medical literature indicates that the surgical treatment of rotator cuff injuries offers a high rate of good and excellent results. ^(^ ^3^ ^)^ In 1987, Ellman et al. introduced arthroscopy as a treatment method that provides greater knowledge of intra-articular lesions and lower morbidity. 

In 2006, 60% of North American surgeons performed repair arthroscopically, a number that rose to 83% a decade later. ^(^ ^2^ Arthroscopic rotator cuff repair constitutes the current standard to treat these injuries in major medical centers around the world. ^(^ ^4^

As with rotator cuff injuries, anterior arthroscopic procedures have also been adopted in place of open ones to treat labral injuries. In 1923, Bankart described labral injuries as the leading cause of recurrent anterior glenohumeral instability of the shoulder, and open repair remained for decades the gold standard to treat this injury, with a recurrence rate below 10%.

Arthroscopic Bankart repair was first described in 1993. The improvement of instruments and surgical techniques has significantly increased the number of surgeries performed by the arthroscopic route, totaling 87.7% of all Bankart repairs performed in the United States in 2008. ^(^ ^5^

Another indicated procedure to treat shoulder instability refers to the Latarjet procedure, which has gained notoriety with the increase in knowledge of the injuries associated with recurrent anterior glenohumeral dislocation and patients’ individual characteristics. Latarjet surgery plays an important role especially in cases of Bankart repair failure and in those patients with significant bone lesions.

However, some shoulder pathologies have no gold standard treatment defined. For example, acromioclavicular dislocations have more than 150 treatment options, leaving surgeons to choose the best technique. ^(^ ^6^ Adequate treatment of such dislocations in their acute phase is critical due to a greater healing potential of the acromioclavicular ligaments. ^(^ ^7^

Rockwood^8^ states that treating acromioclavicular dislocations according to severity into two fundamental options: conservative treatment for Rockwood grade I and II lesions (the greatest controversy occurs for grade III lesions, when each case is evaluated according to patient’s characteristics) and surgical treatment for grade IV, V, and VI lesions. ^(^ ^7^

This study aims to show how SBCOC surgeons currently treat these shoulder pathologies and how they would address them in an ideal scenario.

## MATERIAL AND METHODS

This study was approved by the Research Ethics Committee of the Pedro Ernesto University Hospital/UERJ on June 16, 2021, under number 4,783,761.

A single intersectional survey was carried out in which 314 orthopedists who were SBCOC members and specialized in shoulder and elbow surgery were interviewed via online questionnaire created using a Google form.

The complete survey consisted of two blocks (current scenario and ideal scenario) with 20 questions each. In the current scenario, surgeons answered how they perform the procedures and what materials they use in their daily routine in Brazil. In the ideal scenario, participants answered what they consider to be the best treatment despite the availability of the instruments and material to be used in each procedure. Respondents could mark more than one answer for each topic. 

The questionnaire addressed the surgical materials used by SBCOC members to treat the main shoulder pathologies, such as rotator cuff injuries, anterior glenohumeral instability, and acromioclavicular dislocations. The specialists who consented to participate in this study were anonymized for their gender, age, and area of activity.

All comparisons between groups were evaluated as univariate analyses. Differences in the distribution of categorical variables were evaluated by the McNemar’s test. A significance level was defined for this study (a p-value, i.e., the statistical error admitted in the analyses, equal to 0.05). Statistical analysis was performed on R, version 4.2.3.

## RESULT

This study divided its questionnaire into two scenarios with the same questions, the first referring to specialists’ current reality routine and the second, to the ideal scenario, i.e., that with unlimited surgical materials.

Among the participants, 96% had repaired rotator cuffs arthroscopically in their daily practice (Table 1). Regarding material, metallic anchors are the most used as the method to fixate rotator cuffs (74%) (Table 2).

**Table 1:** Treatment options for rotator cuff repair

	Current (n = 314)	Ideal (n = 314)	p-value
Arthroscopic, n (%)	300 (96%)	301 (97%)	<0.001
Open, n (%)	41 (13%)	20 (6.5%)	<0.001
No answer, n	3	4	

Source: prepared by the authors.

**Table 2:** Treatment options in arthroscopic rotator cuff repair.

	Current (n = 314)	Idea (n = 314)	p-value
Metallic anchors, n (%)	232 (74%)	131 (42%)	0.012
Bioabsorbable anchor, n (%)	138 (44%)	217 (70%)	0.005
Transosseous suture, n (%)	29 (9.2%)	23 (7.4%)	< 0.001
No answer, n	0	3	

Source: prepared by the authors.

In the ideal scenario, most participants also considered arthroscopy the best method (97%), preferring bioabsorbable anchors to repair rotator cuffs. (70%) (p = 0.005).

In the case of open rotator cuff repairs, surgeons prefer to use anchors instead of transosseous sutures in the current (75%) and ideal (79%) scenarios (p< 0.001) (Table 3).

**Table 3:** Treatment options in open rotator cuff repair.

	Current (n = 314)	Ideal (n = 314)	p-value
Anchors, n (%)	212 (75%)	225 (79%)	<0.001
Transosseous Suture, n (%)	111 (39%)	95 (34%)	<0.001
No answer, n	30	30	

Source: prepared by the authors.

In the arthroscopic treatment of glenohumeral instability, most surgeons (87%) chose bioabsorbable anchors in their routine (p< 0.001), as in the ideal scenario, in which 96% of them also deem bioabsorbable anchors as the best fixation method (p < 0.001) (Table 4).

**Table 4:** Material options in the Latarjet procedure.

	Current (n = 314)	Ideal (n = 314)	p-value
Cannulated screw, n (%)	175 (57%)	207 (68%)	<0.001
Non-cannulated screw, n (%)	143 (47%)	109 (36%)	0.005
Cortical screw, n (%)	1 (0.3%)	0 (0%)	<0.001
Cancellous screw, n (%)	1 (0.3%)	1 (0.3%)	<0.001
No answer, n	9	9	

Source: prepared by the authors.

This research also evaluated the number of used anchors, finding similar answers for both scenarios. In daily practice, most members of our Society use three or more anchors (86%) for labral repairs. In the ideal scenario, the preference for three or more anchors increased to 94% of interviewees (p< 0.001) (Table 5).

**Table 5:** Material options in arthroscopic Bankart repair.

	Current (n = 314)	Ideal (n = 314)	p-value
Bioabsorbable anchor, n (%)	271 (87%)	298 (96%)	<0.001
Metallic anchor, n (%)	58 (19%)	17 (5.5%)	<0.001
No answer, n	1	4	

Source: prepared by the authors.

Regarding the treatment for anterior shoulder instability by the Latarjet procedure, SBCOC members prefer cannulated screws (57%) over non-cannulated ones (47%) to fixate the graft to the glenoid in the current scenario. In an ideal scenario, 68% of these professionals would use cannulated screws for graft fixation (Table 6).

**Table 6:** Number of anchors in arthroscopic Bankart repair.

	Current	Ideal	p-value
1-2 Anchors, n (%)	39 (14%)	18 (6.4%)	<0.001
3-4 Anchors, n (%)	249 (86%)	264 (94%)	<0.001
No answer, n	26	32	

Source: prepared by the authors.

This study found that 88% of interviewees treat acromioclavicular dislocations in an open conventional manner in their daily practice (Table 7). Currently, high-strength wires are the most chosen materials (70%), followed by anchors (65%) and Kirschner wires (61%). In the ideal scenario, the open technique remains the best option among specialists (77%), but they also included the endobutton fixation system in their options for surgical materials (54%). Participants chose high-strength wires the most in the ideal scenario, totaling 70% (Table 8).

**Table 7:** Arthroscopic repair for acromioclavicular dislocation: yes or no.

	Current, N= 314	Ideal, N= 314	p-value
Arthroscopic, n (%)	38 (12%)	70 (23%)	<0.001
No answer, n	4	9	

Source: prepared by the authors.

**Table 8:** Material options in the treatment of acromioclavicular dislocation.

	Current, (n = 314)	Ideal, (n = 314)	p-value
Anchors, n (%)	199 (65%)	171 (56%)	<0.001
Endobutton, n (%)	81 (27%)	164 (54%)	<0.001
Kirschner wires, n (%)	186 (61%)	151 (50%)	0.093
High-strength wires, n (%)	213 (70%)	215 (70%)	<0.001
No answer, n	9	9	

Source: prepared by the authors.

## DISCUSSION

### Rotator cuff

Rotator cuff repair has significantly evolved over the past decade due to the advent of less invasive techniques. A study carried out in the United States observed a 600% increase in the number of performed arthroscopies from 1996 to 2006.^9^ This increase in video-assisted surgeries may be related to clinical outcomes similar to open repair and is associated with lower morbidity.

Most SBCOC members also arthroscopically repair rotator cuffs (96%).

A comparative study of the two techniques found better short-term recovery in arthroscopic repairs and equivalent long-term outcomes. An analysis of 1,962 cases showed that the single most important factor in the clinical outcome of rotator cuff repair referred to the initial size of the lesion, ^(^ ^10^ finding no significant differences after analyzing the incidence of complications such as tendon re-rupture and comparing the two techniques. ^(^ ^11^

A biomechanical study on the pullout force of fixation devices considering device types (metal or bioabsorbable anchors), fixation method (transosseous suture or anchors), and use of high-strength threads showed that transosseous sutures have lower pullout resistance than anchors. Comparisons of metallic and bioabsorbable anchors showed similar pullout forces. The use of high-strength sutures instead of conventional ones show no better results in transosseous suturing due to failures in the bone bridge, unlike with anchors, in which high-strength sutures have been shown to increase the strength of the set. ^(^ ^12^

Most participants reported using metallic anchors in their daily practice as they guarantee a secure and long-term fixation. ^(^ ^13^ However, this device increases the technical difficulty in revision surgery^14^ and hinders the interpretation of postoperative MRI, if necessary. ^(^ ^15^ On the other hand, bioabsorbable anchors avoid the potential risks and future difficulties of metallic anchors, which explains the preference for this type of implant. However, they also have disadvantages such as unwanted biological responses, shorter fixation time, and higher cost. ^(^ ^16^

Bioabsorbable implants showed excellent clinical results, resembling those obtained with non-absorbable devices. ^(^ ^17^

### Instability

Glenohumeral instability offers a challenge due to the variety of associated injuries and possible treatments to stabilize the joint. The ISIS score has been developed to help shoulder surgeons define treatment for each case. ^(^ ^18^ A recent multicenter study shows a high reliability rate when using an ISIS score at a cutoff point < 3 (which would indicate arthroscopic repairs).

Bankart open repair has a high success rate, failing only 2% of the time. ^(^ ^19^ With the emergence of arthroscopy, the possibility of performing less invasive procedures has changed the way most shoulder surgeons approach glenohumeral instability, reaching a rate of 90% of repairs performed arthroscopically in 2012. ^(^ ^20^

The reasons for the high adherence of arthroscopy in treating this pathology include its ability to evaluate and treat concomitant lesions that may put Bankart repair at risk alone. ^(^ ^21^ In labral lesions without significant bone lesions, the objective is to fix the capsulolabral complex in its original anatomical position to restore the static restrictor function of the anterior band of the inferior glenohumeral ligament.

Bankart repair should consider the number of anchors to be used. A systematic review on the recurrence rate of instability showed a 15% average recurrence with the use of less than three anchors and one below 10% using three or more anchors. ^(^ ^22^ Comparing these findings with the data in this research, in which the use of one or two anchors represented only 14% of experts’ responses, shows that participants’ choices agree with the literature.

This same review by Brown et al. found no significant difference between the recurrence rate of instability in relation to the type of used anchor (metallic or bioabsorbable). ^(^ ^22^ This study found a more prominent use of bioabsorbable anchors than metallic ones (87% vs. 19%), probably because this intra-articular lesion offers a high risk of complications in case of poor positioning of the metallic anchor.

Labral lesions with significant bone loss (ISIS score above three) require the concomitance of other procedures such as the Remplissage technique or a bone block using the Latarjet technique. ^(^ ^23^

The Latarjet technique is a safe procedure with a low recurrence rate of instability. ^(^ ^24^ A cadaveric biomechanical study evaluated the main methods of fixation of the coracoid graft in the glenoid: unicortical or bicortical, use of cannulated or solid screws, with partial or total thread. It observed no statistical difference in fixation failure between the tested devices. ^(^ ^25^ In Brazil, the members of the SBCOC reported using mostly cannulated screws (57%).

### Acromioclavicular Dislocation

The literature has described about 151 techniques, including the primary repair of acromioclavicular ligaments, reinforcement with autologous grafts, reinforcement with absorbable or non-absorbable sutures, and coracoclavicular stabilization with metal screws. ^(^ ^26^

Historically, metallic devices to fixate the acromioclavicular joint have fallen into disuse due to their greater number of complications and the greater morbidity of the procedure. ^(^ ^27^ More anatomical and less invasive methods have become more popular.

These more anatomical procedures include suspensory techniques, which can use autologous grafts or allografts (usually with a semitendinosus tendon) or high-strength wires fixating the clavicle to the coracoid process by anchors or endobuttons. ^(^ ^28^ The latter cause lower morbidity by requiring neither donor site (in the case of autologous grafts) nor tissue bank availability (as in the case of allografts).

A biomechanical study comparing native coracoclavicular ligaments with TightRope (Arthrex, Naples, Florida) showed that reconstruction with this device obtained a resistance equal or superior to that of native ligaments, evincing a safe fixation method. ^(^ ^29^

A randomized comparative study analyzed clinical outcomes between open and arthroscopic surgeries in treating acute acromioclavicular dislocation, obtaining good results in both procedures. ^(^ ^30^

This study observed that 88% of its specialists prefer open surgeries than arthroscopic ones, and the high-strength wires (70%) as the fixation method.

## CONCLUSIONS

The current Brazilian scenario has a limited availability of instruments and materials SBCOC specialists would prefer in an ideal scenario to treat various shoulder pathologies.

The most recent medical literature corroborates the options SBCOC members chose more often.
